# Breastfeeding Self-Efficacy of Early Postpartum Mothers in an Urban Municipality in the Philippines

**DOI:** 10.31372/20190404.1023

**Published:** 2020

**Authors:** Artemio Morado Gonzales Jr

**Affiliations:** a Occidental Mindoro State College, Philippines

**Keywords:** breastfeeding, self-efficacy, BSES-SF, postpartum

## Abstract

Studies on women have identified breastfeeding confidence as an important variable in influencing breastfeeding outcomes. The mother’s breastfeeding self-efficacy in the early postpartum period was a strong predictor of the duration of breastfeeding. This study aims to assess the breastfeeding self-efficacy of the postpartum mothers in urban barangays of San Jose Occidental Mindoro.

The respondents of the study were 200 early postpartum mothers distributed equally chosen from four purposively selected urban barangays. The data collection technique was through a survey interview using 14-item Breastfeeding Self-Efficacy Scale-Short Form (BSES-SF) and a demographic questionnaire. Descriptive and inferential statistics were used to analyze the data.

The study revealed that postpartum mothers responded in the study were confident and has self-efficacy in breastfeeding their child as measured through technique and intrapersonal thought in breastfeeding. Moreover, the number of prenatal check up was positively correlated with breastfeeding self-efficacy.

The result of the study can be used as a baseline assessment tool in the hospital at delivery to assist in identifying women who are at risk for early weaning.

## Introduction

Despite the significant health advantages of breastfeeding, including both in the short term and the longer term to infants and their mothers (Binns, Lee, & Low, 2016), not all women initiate breastfeeding. Based on the recommendation of the American Academy of Pediatrics (2005) and World Health Organization (2003) optimal breastfeeding includes early initiation of breastfeeding, exclusive breastfeeding for the first 6 months, and continued breastfeeding for at least 2 years while adding appropriate complementary foods. Breastfeeding is sufficient and beneficial for infant nutrition in the first 6 months of life (Ip, Chung, Raman, Trikalinos, & Lau, 2009; Prell & Koletzko, 2016). Breastfeeding immediately after birth also helps the uterus contract, hence reducing the mother’s postpartum blood loss. Giving any other foods and water (in addition to breast milk) before the child in age 6 months is discouraged because it may inhibit breastfeeding and expose the infant to illness. Infants older than 6 months need other food and drink while they continue to breastfeed until age 2 or older. Breastmilk is an important source of energy, protein, and other nutrients such as vitamin A and iron (PAHO/WHO, 2003).

According to the most recent Family Health Survey, 92% of children in the Philippines aged 6–35 months old had been breastfed at some time but only 27% were exclusively breastfed. A higher percentage of poor children (34%) than non-poor children (24%) were exclusively breastfed. MIMAROPA Region has been below the national threshold performing 36.7% (Philippines Statistics Authority, 2014). The Infant and Young Child Feeding (IYCF) Plan of Action (2011–2016) has set a target of 90% breastfeeding rate by the end of 2016. Moreover, it also targeted that 90% of the newborn should initiate breastfeeding within 1 hour after childbirth and 70% exclusive breastfeeding up to 6 months. The same survey revealed that almost half of all pregnant women deliver at a health facility, while half deliver at home (World Health Organization, 2015). Furthermore, the identified problem among breastfeeding mothers was the disconnection among breastfeeding initiation rates in the hospital, breastfeeding exclusivity upon discharge, and breastfeeding rates at 2 weeks postpartum. Two weeks postpartum is a well-documented time period for breastfeeding cessation (Glassman, McKearney, Saslaw, & Sirota, 2014; Hackman, Alligood-Percoco, Martin, Zhu, & Kjerulff, 2016).

Maternal self-efficacy is defined as a mother’s perceived ability to breastfeed her child and influence her decisions regarding breastfeeding, such as whether to breastfeed, how much effort to place on breastfeeding, and how to respond to any challenges during the experience (Dennis, 1999). Studies on women have identified breastfeeding confidence as an important variable in influencing breastfeeding outcomes (Hadjiona et al., 2016; Loke & Chan, 2013; Newby & Davies, 2016). It was agreed by Lau, Lok and Tarrant (2018) saying the constructs of the Theory of Reasoned Action (TRA), Theory of Planned Behavior (TPB), and Breastfeeding Self-Efficacy (BSE) Framework can effectively identify relationships between maternal psychosocial factors and breastfeeding initiation. However, inconsistent findings were found in assessing the relationship between maternal attitudes, subjective norms, perceived behavior control, and breastfeeding duration. The inadequacy of these constructs in explaining breastfeeding duration indicates a need to further explore the role of maternal self-determination in breastfeeding behavior. Further, it was revealed that studies pertaining to breastfeeding self-efficacy in Asia mostly concentrates in Turkey (Alus Tokat, Serçekuş, Yenal, & Okumuş, 2015), Iran (Mirghafourvand, Kamalifard, Ranjbar, & Gordani, 2018), China (Wu, Hu, McCoy, & Efird, 2014; Yang, Gao, Ip, & Sally Chan, 2016), Hong Kong (*n* = 3), Cyprus (Hadjiona et al., 2016), Jordan (Abuidhail, Mrayan, & Jaradat, 2019), Saudi Arabia (Khresheh & Ahmad, 2018), Taiwan (Lee, Chang, & Chang, 2019), and Vietnam (Ngo, Chou, Gau, & Liu, 2019). Little is known about the breastfeeding self-efficacy and sociocultural factors in the early postpartum period among low-income Filipino.

Breastfeeding support extends beyond nurses, physicians, and significant others as they play a significant role in the promotion of breastfeeding (Almeida, Luz, & Ued, 2015; Brown & Davies, 2014). However, despite all of the breastfeeding education available from healthcare professionals, the true test of breastfeeding support and success lies within the social support to increase breastfeeding self-efficacy. The purpose of this paper is to assess the breastfeeding efficacy of the postpartum mothers occurring over 2–6 weeks in an urban municipality in Occidental Mindoro.

## Methods

### Research Design

The research design of the study was descriptive-correlational to determine the level of breastfeeding self-efficacy of the postpartum mothers occurring over 2–6 weeks in urban barangays in San Jose, Occidental Mindoro. This study was conducted from November 2017 to January 2018.

### Study Site

The study was conducted in selected barangays of San Jose, Occidental Mindoro that comprised the large populace barangays and with a high birth rate namely San Roque, Pag-asa, Caminawit, and Labangan. San Jose is a municipality Mindoro in the seventh largest island in the Philippines. The majority of the population consists of a mix of migrants of different ethnolinguistic groups from nearby provinces, namely Tagalogs, Bicolanos, Visayans, Kapampangans, Pangasinans, and Ilocanos.

### Sample

The study assumes a 95% confidence interval with a *Z* value of 1.96 and a margin of error of 5%. With this, the estimated sample size in this study should have 246 postpartum mothers (occurring over 2–6 weeks postpartum). The respondents of the study were 200 early postpartum mothers distributed equally chosen from four purposively selected barangays: San Roque, Pag-asa, Caminawit, and Labangan. For each selected areas, respondents were chosen using systematic sampling. The study has an 81% response rate.

### Research Instrument

The questionnaire was composed of two parts: the socio-demographic section and the breastfeeding self-efficacy scale. The socio-demographic section includes age (ratio), civil status (nominal), educational attainment (ordinal), income level (ratio), gravida (nominal), parity (nominal), number of prenatal visit, place of delivery (nominal), attendant during childbirth (nominal), and breastfeeding status (nominal). The second part of the questionnaire was the 14-item Breastfeeding Self-Efficacy Scale-Short Form (BSES-SF) by Dennis (2002). The BSES-SF is a self-report instrument, containing two subscales: (1) the technique subscale, where items depict maternal skills and recognition of specific principles required for successful breastfeeding; and (2) the intrapersonal thoughts subscale, where items are related to maternal attitudes and beliefs towards breastfeeding. Items are preceded by the phrase “I can always” and anchored with a 5-point Likert scale, where 1 means not at all confident and 5 means always confident. As recommended (Bandura, 1977), all items are presented positively, and scores are summed to produce a range from 14 to 70, with higher scores indicating higher levels of breastfeeding self-efficacy.

A study provided preliminary evidence that the BSES-SF may be an internationally applicable, reliable, and valid measure to assist health professionals in caring for breastfeeding women. Cronbach’s alpha coefficient for internal consistency was .80 and above (Tuthill, McGrath, Graber, Cusson, & Young, 2016).

### Data Collection

The data collection technique was through a survey interview using a questionnaire. The postpartum mothers were approached during scheduled first postpartum and expanded program for immunization clinic visits in the barangay health center. Informed consent was attained from the mothers before the researchers conducted the interview.

### Ethical Consideration

This paper was technically reviewed and approved by the Research Council of the Occidental Mindoro State College under its Research Development and Extension Unit. Participation in the study was voluntary and explained to the mothers that they have the option not to answer the questionnaire or not. Complete anonymity of the research participants was observed. The respondents were informed of the right to confidentiality and privacy. Any clarifications were entertained by the researcher to facilitate easy understanding of the statement in the research instrument. The questionnaire was coded and listed in a separate sheet, the code from the list was later matched after data collection. Specific information on the questionnaires could not be linked to specific individuals. Access to the data was limited only to the researcher.

### Data Analysis

Frequency and percentage were computed to describe the demographic profile such as age, civil status, educational attainment, income level, gravida, parity, the number of prenatal visits, place of delivery, and attendant at birth. Despite the previous recommendation of Bandura (1977), the breastfeeding self-efficacy scores for each item were analyzed using weighted mean and grouped in either: highly confident (4.20–5.00), confident (3.40–4.19), moderately confident (2.60–3.39), not confident (1.80–2.59), and highly not confident (1.00–1.79) to interpret the level of confidence on a mean factor. In this process of quantification, it permits the contents (items) in such scales/models to be rather flexible and need-based. The analysis was made for the two subscales of breastfeeding self-efficacy separately using Kendall’s tau. The outcome variable was the level of breastfeeding self-efficacy while the explanatory variables were socio-demographic and related variables. Finally, a correlational analysis was conducted to determine the association between breastfeeding self-efficacy and socio-demographic characteristics. A *p*-value of .05 was taken for statistical significance.

## Results

### Profile of the Postpartum Mothers in the Study

Data presented in Table 1 shows that the mean age of the respondents was 27.4 ± 6.9 years. This suggests that majority of the respondents were at their adult age. It also reveals that the respondents were cohabiting (51.5%), reached high school (51.5%), earning 1,000–5,000 (40.5%), multigravid (72.0%), and multiparous (73.0%). In terms of access in healthcare during pregnancy and delivery, most of the respondents had a prenatal visit of 4 and above (88.5%), gave birth in a government hospital (35.5%), and handled by a midwife (47.5%). The respondents were mostly having exclusive breastfeeding (56.5%).

**Table 1:** Profile of the Respondents

Profile	Frequency	Percentages
Age	mean ± SD = 27.4 ± 6.9	
Marital status		
Single	11	5.5
Cohabiting	103	51.5
Married	86	43.0
Educational attainment		
No formal education received	2	1.0
Elementary	25	12.5
High school	103	51.5
College	70	35.0
Monthly income		
1,000–5,000	81	40.5
6,000–10,000	74	37.0
11,000–20,000	31	17.0
20,000 and above	11	5.5
Gravida		
Primigravid	56	28.0
Multigravid	144	72.0
Parity		
Primiparous	54	27.0
Multiparous	146	73.0
Number of prenatal visit		
Less than 4	23	11.5
4 and above	177	88.5
Place of delivery		
Home	25	12.5
BEmONC facility	30	15.0
Accredited lying - in	63	31.5
Government hospital	71	35.5
Private hospital	11	5.5
Attendant during childbirth		
Hilot	25	11
Nurse	1	0.5
Midwife	95	47.5
Doctor	79	39.5
Infant feeding style		
Exclusive bottle-feeding	13	6.5
Mixed feeding	74	37.0
Exclusive breastfeeding	113	56.5

### Breastfeeding Self-Efficacy of the Respondents

Table 2 revealed that postpartum mothers responded in the study were confident and has self-efficacy in breastfeeding their child (mean = 4.01). It was also revealed that the respondents were both confident in breastfeeding technique (mean = 3.90) and intrapersonal thought in breastfeeding (mean = 4.12).

**Table 2:** Breastfeeding Self-Efficacy Scores of the Respondents

BSE subscale	Mean	Interpretation
**Technique**		
1. I can always determine that my baby is getting enough milk	3.87	Confident
2. I can always ensure that my baby is properly latched on for the whole feeding	3.68	Confident
3. I can always manage the breastfeeding situation to my satisfaction	4.54	Highly confident
4. I can always manage to breastfeed even if my baby is crying	3.40	confident
5. I can always comfortably breastfeed with my family members present	4.48	Highly confident
6. I can always deal with the fact that breastfeeding can be time-consuming	3.85	confident
7. I can always finish feeding my baby on one breast before switching to the other breast	3.76	Confident
8. I can always manage to keep up with my baby’s breastfeeding demands	3.79	Confident
9. I can always tell when my baby is finished breastfeeding	3.73	Confident
**Weighted mean**	**3.90**	**Confident**
**Intrapersonal thoughts**		
1. I can always successfully cope with breastfeeding as I have with other challenging tasks	3.94	Confident
2. I can always breastfeed my baby without using formula as a supplement	3.96	Confident
3. I can always keep wanting to breastfeed	4.31	Highly confident
4. I can always be satisfied with my breastfeeding experience	4.59	Highly confident
5. I can always continue to breastfeed my baby for every feeding	3.79	Confident
**Weighted mean**	**4.12**	**Confident**
**Overall breastfeeding self-efficacy**	**4.01**	**Confident**

Highly confident (4.20–5.00); confident (3.40–4.19); moderately confident (2.60–3.39); not confident (1.80–2.59); and highly not confident (1.00–1.79).

### Respondent’s Profile and Breastfeeding Efficacy

The result shows that it is only the number of prenatal checkup that is positively correlated with confidence on breastfeeding technique (*r* = .183, *p*-value = .010), intrapersonal thoughts in breastfeeding (*r* = .225, *p*-value = .001), and overall breastfeeding efficacy (*r* = .207, *p*-value = .003) as shown in Table 3. It means that as the number of prenatal checkup increases, the breastfeeding self-efficacy also increases.

**Table 3:** Correlation between Respondent’s Profile and Breastfeeding Self-Efficacy

Profile	Technique		Intrapersonal thoughts		Overall BSES	
	*r*	*p*	*r*	*p*	*r*	*p*
Age	.08	.264	.04	.587	.61	.393
Marital status	.04	.552	.04	.596	.04	.563
Educational attainment	−.80	.258	−.129	.068	−.106	.134
Monthly income	−.047	.512	−.037	.607	−.042	.558
Gravida	.101	.156	.104	.141	.104	.142
Parity	.093	.189	.098	.169	.097	.171
Number of prenatal visit	.183	.010^∗^	.225	.001^∗^	.207	.003^∗^
Place of delivery	.028	.696	.027	.707	.028	.699
Attendant during childbirth	.025	.730	.012	.866	.007	.925
Infant feeding style	.006	.935	.005	.945	.001	.985

Significant at *p* ≤ 0.05.

## Discussion

Majority of the respondents were at their early adult age. According to the Philippine Statistics Authority (2014), fertility peaks at age 20–24 and falls after 25–39. The findings on the current study also suggest that the majority of them did not pursue at aiming the highest level of formal education. In the study of Oikawa et al. (2014), it was revealed that educated women are more likely to use maternal care services than women with no formal education. According to the Philippine Statistics Authority, the national poverty threshold in 2015 is 10,969 per month. Poverty threshold includes basic non-food needs such as clothing, housing, transportation, health, and education expenses (PSA, 2016). This indicates that majority of the respondents were below the poverty threshold. Populations who belong to low-income family could hardly afford to subject themselves to take the recommendations required for health improvement due to economic status (Bircher & Hahn, 2017). Results of the study show that there is a low contraceptive prevalence rate in terms of family planning; this was based on the history of pregnancies and parity revealed by the respondents. With regards to prenatal checkup during their pregnancy, the finding was supported by the Department of Health (2012) statement that the Department of Health recommends a minimum of four (4) visits for the whole course of pregnancy. A pregnant woman has at least one visit for the first and second trimester and two visits for the third trimester. The quality of prenatal care is an important indicator of maternal and infant health status (Heredia-Pi, Servan-Mori, Darney, Reyes-Morales, & Lozano, 2016). If a mother is equipped with adequate knowledge in prenatal care, she is most likely to comply with the prenatal checkup and habits to attain maximum health during pregnancy, childbirth, and puerperium. The better result is attained with regards to women with facility-based deliveries and handled by skilled birth attendants.

The results from the current study are consistent with the original BSES-SF study of Dennis (2010) and provide evidence that the BSES-SF is a reliable measure of breastfeeding self-efficacy among a representative sample in San Jose, Occidental Mindoro. The study investigated the effects of behavior choices, effort, persistence, thought patterns, and emotional reactions of breastfeeding women to establish a connection between confidence levels and response to external and internal factors. The study discovered that self-efficacy was affected by all of these components and predicted initiation, performance, and maintenance of breastfeeding activities. However, it contradicts the findings of Newby and Davies (2016) which states that there is anticipated social discomfort breastfeeding in public and significantly more likely to anticipate discomfort breastfeeding in the presence of close female friends. Moreover, Brandao et al. (2017) depicted that breastfeeding self-efficacy can help identify pregnant women at higher risk to prematurely discontinuing breastfeeding and who may require additional intervention from health providers to ensure breastfeeding success.

The study revealed that as the number of prenatal checkup increases, the breastfeeding self-efficacy also increases. It could be possibly explained by the finding of Yurtsal and Kocoglu (2015) stating that the rates for the mothers’ early initiation of breastfeeding, exclusive breastfeeding continuation rates, and the mean scores the participants obtained from mean scale scores increased when they were given training and counseling on breastfeeding from the prenatal period until the end of the first 6 months postpartum. The result of the current study contradicts the findings of Hinic (2016) which states that breastfeeding self-efficacy was positively correlated with birth satisfaction, number of children, partner support of breastfeeding, intention to breastfeed, intention to breastfeed exclusively for 6 months, and feeling prepared for birth. Breastfeeding self-efficacy was greater in women with previous breastfeeding experience and lower in mothers of newborns who received in-hospital formula supplementation. On the other hand, it corroborates with the study conducted by Dodt, Ximenes, Almeida, Oriá, and Dennis (2012) revealing no relationship was found between breastfeeding self-efficacy and maternal occupation, educational level, marital status, family income, or the number of pregnancy suggesting BSES-SF may be a unique tool to identifying women at risk to prematurely discontinue breastfeeding. But the same study also revealed that a significant relationship was found between breastfeeding self-efficacy and maternal age. The contradiction was also supported by Yang et al. (2016) stating that age, education, occupation, monthly household income, time of the decision to breastfeed, and delivery mode are not to be proven as a predictor of breastfeeding self-efficacy.

A limitation of this study was the fact that the sample of the study was composed of postpartum women who presented to outpatient clinics. This research does not claim findings representative of all Filipino women. It is difficult to state that the sample used fully represented the sociocultural groups living in the province. It is important to conduct further studies to test the psychometric properties of the scale in samples representing different groups. Another limitation in this study was limited by its cross-sectional nature, as a result of which the relationships between breastfeeding self-efficacy and socio-demographic variables do not necessarily indicate causal relationships.

## Conclusions

The respondents in the study are literate, living below the poverty line, and young adults. They met the required number of prenatal visits, utilized accredited birthing facilities, and handled by skilled birth attendants. Furthermore, the respondents mostly practiced exclusive breastfeeding. The postpartum mothers responded in the study were confident in breastfeeding as a measure of breastfeeding efficacy. The number of prenatal checkup is positively correlated with breastfeeding self-efficacy which means as the number of prenatal checkup increases, the breastfeeding self-efficacy also increases.

## Recommendations

The results of this study can be used as a baseline assessment tool in the hospital at delivery to assist in identifying women who are at risk for early weaning as evidenced by low breastfeeding self-efficacy. Strenuous public health efforts are needed to improve breastfeeding behaviors, particularly among socioeconomically disadvantaged groups. Caregivers need to fully understand the expectations that patients have in their care and provide care that is consistent with those expectations. Further research is necessary to examine how certain interventions may help foster self-efficacy and thus breastfeeding duration.

## Acknowledgments

The author thanks all the women participated in the study and the Occidental Mindoro State College for administrative support. Further, thanks were also given to the midwives Rosavelle Hayag, Merlinia Panganiban, Laurensa Joldanero, and Menchie Tayanan for the assistance during the data collection.

## Declaration of Conflicting Interests

The authors declared no potential conflicts of interest with respect to the research, authorship, and/or publication of this article.

## Funding

The author acknowledges receipt of financial and administrative support from Occidental Mindoro State College through Research, Development and Extension Unit.
